# Retained differentiation capacity of human skeletal muscle satellite cells from spinal cord‐injured individuals

**DOI:** 10.14814/phy2.13739

**Published:** 2018-06-14

**Authors:** Mladen Savikj, Maxwell A. Ruby, Emil Kostovski, Per O. Iversen, Juleen R. Zierath, Anna Krook, Ulrika Widegren

**Affiliations:** ^1^ Faculty of Medicine University of Oslo Oslo Norway; ^2^ Science Department Sunnaas Rehabilitation Hospital Nesoddtangen Norway; ^3^ Department of Physiology and Pharmacology Karolinska Institutet Stockholm Sweden; ^4^ Department of Molecular Medicine and Surgery Karolinska Institutet Stockholm Sweden; ^5^ Department of Nutrition and Preventive Medicine Norwich Medical School University of East Anglia Norwich Norfolk United Kingdom; ^6^ Institute of Clinical Medicine University of Oslo Oslo Norway; ^7^ Department of Nutrition Institute of Basic Medical Sciences University of Oslo Oslo Norway; ^8^ Department of Haematology Oslo University Hospital Oslo Norway

**Keywords:** Muscle atrophy, satellite cells, skeletal muscle, spinal cord injury

## Abstract

Despite the well‐known role of satellite cells in skeletal muscle plasticity, the effect of spinal cord injury on their function in humans remains unknown. We determined whether spinal cord injury affects the intrinsic ability of satellite cells to differentiate and produce metabolically healthy myotubes. We obtained *vastus lateralis* biopsies from eight spinal cord‐injured and six able‐bodied individuals. Satellite cells were isolated, grown and differentiated in vitro. Gene expression was measured by quantitative PCR. Abundance of differentiation markers and regulatory proteins was determined by Western blotting. Protein synthesis and fatty acid oxidation were measured by radioactive tracer‐based assays. Activated satellite cells (myoblasts) and differentiated myotubes derived from skeletal muscle of able‐bodied and spinal cord‐injured individuals expressed similar (*P* > 0.05) mRNA levels of myogenic regulatory factors. Myogenic differentiation factor 1 expression was higher in myoblasts from spinal cord‐injured individuals. Desmin and myogenin protein content was increased upon differentiation in both groups, while myotubes from spinal cord‐injured individuals contained more type I and II myosin heavy chain. Phosphorylated and total protein levels of Akt‐mechanistic target of rapamycin and forkhead box protein O signalling axes and protein synthesis rate in myotubes were similar (*P* > 0.05) between groups. Additionally, fatty acid oxidation of myotubes from spinal cord‐injured individuals was unchanged (*P* > 0.05) compared to able‐bodied controls. Our results indicate that the intrinsic differentiation capacity of satellite cells and metabolic characteristics of myotubes are preserved following spinal cord injury. This may inform potential interventions targeting satellite cell activation to alleviate skeletal muscle atrophy.

## Introduction

Traumatic spinal cord injury has a prevalence of between 250 and 906 cases per million in developed countries (Singh et al. 2014), and leads to severe physical and psychosocial consequences. It is characterized by varying degrees of motor, sensory and autonomic neurological deficits below the level of injury, affecting most bodily systems (Binder 2013). Decentralization of skeletal muscle from the nervous system causes inactivity and marked atrophy, with a decrease of both single fiber and whole muscle cross sectional area (Castro et al. 1999; Gorgey and Dudley 2007). The decrease in muscle mass is attributed to an imbalance between protein synthesis and degradation, with associated changes in the protein kinase B (Akt) – mechanistic target of rapamycin (mTOR) signalling axis, and activity of the forkhead box protein O (FoxO) transcription factors and their targets, respectively (Jackman and Kandarian 2004; Dreyer et al. 2008; Leger et al. 2009). Atrophy reduces the size of all fibers and shifts the fiber type composition from type I oxidative fibers to predominantly type IIx glycolytic fibers (Lotta et al. 1991; Aksnes et al. 1996; Castro et al. 1999; Kostovski et al. 2013). Along with morphological changes, spinal cord injury reduces the fatty acid oxidation capacity of skeletal muscle (Wang et al. 1999; Kjaer et al. 2001; Long et al. 2011; McCully et al. 2011). Moreover, the reduced muscle mass diminishes peripheral glucose disposal (Aksnes et al. 1996). Impairments in both lipid and glucose metabolism ultimately increase the risk of noncommunicable diseases such as type 2 diabetes and cardiovascular disease (Cragg et al. 2013a,b).

Satellite cells are located between the sarcolemma and the basement membrane of the muscle fibers (Mauro 1961). These cells play an integral role in muscle plasticity and regeneration through self‐renewal and fusion into the existing fibers (Schiaffino et al. 1976; Collins et al. 2005; Bruusgaard et al. 2010; Lepper et al. 2011). Paired box protein 7 (Pax7) positive satellite cells express myogenic regulatory transcription factors, which show commitment to the myogenic lineage (e.g., myogenic factor 5 – Myf5 and myogenic differentiation factor 1 – Myod1) and passage into terminal differentiation (e.g. myogenin) (Almeida et al. 2016).

Individuals with spinal cord injury have a reduced number of satellite cells per skeletal muscle fiber (Verdijk et al. 2012). It is yet unclear whether spinal cord injury affects the differentiation capacity of human skeletal muscle satellite cells. Altered function of satellite cells has been indicated by animal models. Spinal cord transection and contusion in rats lead to satellite cell activation (Dupont‐Versteegden et al. 1999; Jayaraman et al. 2013). However, the differentiation of satellite cells may be lacking, as the myonuclear number continues to decrease in these animals in spite of satellite cell activation (Dupont‐Versteegden et al. 1999). Several types of skeletal muscle atrophy, with an underlying neurological mechanism, are accompanied with abnormal satellite cell differentiation. Denervation of rat skeletal muscle leads to formation of new myotubes with defective contractile machinery (Carraro et al. 2015). Skeletal muscle satellite cells from individuals with amyotrophic lateral sclerosis have reduced differentiation capacity and form myotubes with abnormal morphology (Pradat et al. 2011; Scaramozza et al. 2014). However, whether skeletal muscle satellite cell differentiation is affected by spinal cord injury in humans remains unknown. As satellite cells play a role in regulating skeletal muscle mass and are responsive to exercise stimuli (Bruusgaard et al. 2010), their ability to differentiate is of importance in efforts to maintain skeletal muscle mass following spinal cord injury.

Here, we determined the effect of spinal cord injury on the intrinsic ability of satellite cells to differentiate. Additionally, we assessed the metabolic properties of cultured myotubes to determine whether changes seen in vivo in skeletal muscle after spinal cord injury are reflected in satellite cell‐derived myotubes in vitro. Myotubes differentiated from satellite cells in vitro retain characteristics of the donor skeletal muscle in several metabolic and neurological conditions (Bouzakri et al. 2004; Pradat et al. 2011; Boyle et al. 2012; Green et al. 2011, 2013; Jiang et al. 2013; Scaramozza et al. 2014). Thus, primary human skeletal muscle cultures have provided meaningful insight into satellite cell function in muscle disorders.

## Materials and Methods

### Study participants

Fourteen individuals were studied, including eight men with a longstanding (more than 1 year) spinal cord injury currently undergoing routine follow‐ups at Sunnaas rehabilitation hospital (Oslo, Norway), and six age‐matched able‐bodied controls. Participants with spinal cord injury received anticoagulant (clexane or equivalent) therapy during the first 3–6 months after injury as well as spasmolytic therapy (baclofen or equivalent). They did not receive any corticosteroid treatment. Six men with no history of smoking, nonathletes, with no current use of medications were recruited as able‐bodied controls. Neither spinal cord‐injured nor able‐bodied participants had any known malignant, systemic or musculoskeletal disease, nor an intercurrent infection. Participant characteristics are shown in Table 1. The study was conducted according to the ethical principles expressed in the declaration of Helsinki (World Medical, A, 2013). All participants gave their written informed consent and the study was approved by the Regional Committee for Medical and Health Research Ethics at Helse Sør‐Øst Trust, Norway.

**Table 1:** Participant characteristics

	Age years (range)	BMI (kg m^−2^; mean ± SEM)
Able‐bodied participants (*n* = 6)	42 (33; 54)	22 ± 1
Spinal cord‐injured participants (*n* = 8)	52 (26; 71)	26 ± 2^1^
Level of injury (*n*)	Motor deficiency	Time since injury (years)
C7 (2)	Complete	11; 4
C7	Incomplete	8
Th4	Complete	34
Th5	Complete	20
Th12 (3)	Incomplete	6; 5; 4

^1^ *P *=* *0.06 for BMI (able‐bodied vs. spinal cord‐injured).

### Skeletal muscle biopsy procedure

Participants were instructed to abstain from physical activity the day before the biopsy. Skeletal muscle samples were obtained by either a semi‐open or by an open biopsy procedure (Henriksson 1979; Berman et al. 1985) from the mid portion of *vastus lateralis* of the *quadriceps femoris* muscle under local anesthesia (Lidocaine 5 mg mL^−1^). A single piece of skeletal muscle (50–100 mg) was obtained by either procedure and immediately transferred to ice cold phosphate buffered saline (PBS) supplemented with 1% PenStrep (100 UI mL^−1^ penicillin and 100 *μ*g mL^−1^ streptomycin) and kept on ice.

### Satellite cell isolation and culture of primary skeletal muscle cells

Satellite cells were isolated from skeletal muscle samples and cultured as previously described (Mudry et al. 2017). Satellite cells were extracted using a collagenase solution; attachment of nonsatellite cells was allowed for 1 h, after which the supernatant containing satellite cells was collected. Cells proliferated at 37°C and 7.5% CO_2_ in growth medium (20% fetal bovine serum – FBS). Differentiation was induced at ~80% confluence, using fusion medium (100 *μ*g mL^−1^ apo‐transferrin and 0.286 IU mL^−1^ insulin) for 4 days. Experiments were performed on differentiated myotubes after an additional 4 days in post‐fusion medium (2% FBS). For assessment of differentiation cells were harvested prior to (myoblasts), and 8 days after (myotubes) the induction of differentiation.

### Immunohistochemistry of primary skeletal muscle cell cultures

Immunohistochemistry was performed as previously described (Sjogren et al. 2015). Myoblasts and myotubes were fixed in 4% paraformaldehyde in PBS for 15 min at room temperature. Desmin and Ki67 were detected by indirect immunofluorescence using primary antibodies against desmin (#15200 Abcam, Cambridge, U.K.) at a concentration of 1:500 and against Ki67 (#9449 Cell Signaling, Danvers, MA) at a concentration of 1:800. Secondary antibodies used (5 *μ*g mL^−1^) were Alexa Fluor 488 (#A‐11008, Invitrogen, Carlsbad, CA) and Alexa Fluor 594 (#A‐11005, Invitrogen). Nuclei were stained by 4,6‐diamidino‐2‐phenylindole (DAPI) (#D1306, Molecular Probes, Eugene, OR) according to the manufacturer's instructions.

### RNA isolation, cDNA synthesis and qPCR

Cells were harvested in TRIzol (#15596‐018, Life Technologies, Carlsbad, CA) and RNA was isolated according to the manufacturer's instructions. RNA concentration was determined by Nanodrop ND‐1000 (Thermo Fischer Scientific) and 1000 ng was loaded as template for cDNA synthesis, which was performed using the high‐capacity cDNA reverse transcriptase kit (#4368814, Applied Biosystems, Foster City, CA). Quantitative PCR (qPCR) was performed using Fast SYBR Green Master Mix (#4385612, Applied Biosystems) and Ct values were determined using StepOne software v2.1 (Applied Biosystems). The results were normalized to the geometric mean of two controls genes, TATA‐binding protein (TBP) and 60S acidic ribosomal protein P0 (RPLP0), and the expression calculated by the ∆Ct method. Primers used for the reactions were designed to exon to exon junctions of the targeted genes. Sequences of used primers are listed in Table 2. Each assay was accompanied by a melt curve step, which have shown a single clear peak for each set of primers.

**Table 2:** Sequences of primers used for qPCR

Target gene	Forward primer	Reverse primer
TBP	AGTTCTGGGATTGTACCGCA	TATATTCGGCGTTTCGGGCA
RPLP0	TGGAGAAACTGCTGCCTCAT	GATTTCAATGGTGCCCCTGG
Pax7	GAGGACCAAGCTGACAGAGG	CTGGCAGAAGGTGGTTGAA
Myf5	CCACCTCCAACTGCTCTGAT	GCAATCCAAGCTGGATAAGG
Myod1	AGCACTACAGCGGCGACT	GCGCCTCGTTGTAGTAGGC
Myf6	GGATCAGCAGGAGAAGATGC	CCTGGAATGATCGGAAACAC
Myogenin	GCTCAGCTCCCTCAACCA	GCTGTGAGAGCTGCATTCG
Desmin	CTGGAGCGCAGAATTGAATC	GGCAGTGAGGTCTGGCTTAG

### SDS‐PAGE and Western blot

Cells were harvested in ice cold lysis buffer (20 mmol L^−1^ Tris‐HCl pH 7.8) containing protease and phosphatase inhibitors (#539131, Protease Inhibitor Coctail Set I – Calbiochem, Merck Millipore, Billerica, MA; 1 mmol L^−1^ PMSF; 0.5 mmol L^−1^ Na_3_VO_4_). Protein concentration was determined using Pierce BCA protein assay kit (#23225, Thermo Fischer Scientific). Equal amounts of protein were diluted in Laemmli buffer. Sodiumdodecyl sulfate polyacrylamide gel electrophoresis (SDS‐PAGE) was performed as previously described (Sjogren et al. 2015), using Criterion XT Bis‐Tris 4–12% precast gels (#3450124, BioRad, Hercules, CA). Protein was transferred to an Immobilion‐P polyvinylidene fluoride (PVDF) membrane (#IPVH00010, Merck Millipore). Ponceau staining was performed and the results are normalized to the total amount of protein per lane. Western blotting was performed using primary antibodies overnight, at a 1:1000 concentration in tris‐buffered saline (TBS) containing 0.1% bovine serum albumin (BSA) and 0.1% NaN_3_. The antibodies used are listed in Table 3. Species‐appropriate horseradish peroxidase conjugated secondary antibodies were used at a concentration of 1:25,000 in 5% milk in TBS‐Tween. Proteins were visualized by chemiluminescence (#RPN2232 ECL and #RPN2235 ECL select Western blotting detection reagent – GE Healthcare, Little Chalfont, U.K.) and the quantification was performed by ImageLab software v. 5.2.1 (BioRad).

**Table 3:** Primary antibodies used for Western blot

Antigen	Molecular weight (kDa)	Product number and supplier
Desmin	55	#15200 Abcam, Cambridge, U.K.
Myogenin	34	#12732 Santa Cruz, Dallas, TX
MHC II (Myh1/2)	225	#53088 Santa Cruz
MHC I (Myh7)	225	#A4.840 DSHB, Iowa City, IA
pAkt^(Ser473)^	60	#4060 Cell Signaling Danvers, MA
pAkt^(Thr308)^	60	#4056 Cell Signaling
Akt	60	#9272 Cell Signaling
pmTOR^(Ser2448)^	289	#600‐401‐422 Rockland, Pottstown, PA
mTOR	289	#2983 Cell Signaling
Raptor	150	#2280 Cell Signaling
p4EBP1^(Thr37/46)^	20	#2855 Cell Signaling
4EBP1	20	#9452 Cell Signaling
pS6^(Ser235/236)^	32	#2211 Cell Signaling
S6	32	#2317 Cell Signaling
pFoxO1^(Ser256)^	78	#9461 Cell Signaling
FoxO1	78	#12161 Abcam
pFoxO3a^(Ser253)^	97	#13129 Cell Signaling
FoxO3a	97	#47409 Abcam
LC3	16/18	#L8918 Sigma Aldrich, St. Louis, MO
p62	62	# P0067 Sigma Aldrich
MAFbx	35	#166806 Santa Cruz
Pan 20S*α*	30–32	#22674 Abcam
pACC^(Ser222)^	280	#3661 Cell Signaling
ACC	280	#3676 Cell Signaling

MHC, myosin heavy chain; mTOR, mechanistic target of rapamycin; 4EBP1, 4E binding protein 1; FoxO, forkhead box protein O; LC, light chain; MAFbx, muscle atrophy F‐box; ACC, acetyl‐CoA carboxylase.

### [^14^C]Phenylalanine incorporation into protein

Fully differentiated myotubes were incubated in Dulbecco's Modified Eagle Medium (DMEM) (1 g L^−1^ glucose) with 425 *μ*mol L^−1^ phenylalanine and 0.4 *μ*Ci mL^−1^ of [^14^C] phenylalanine (#NEC284E050UC, Perkin Elmer, Waltham, MA) for 6 h at 37°C and 7.5% CO_2_. The cells were then washed in PBS and lysed in 0.03% SDS for 1 h at room temperature. The total amount of protein per well was determined by the Pierce BCA protein assay kit (#23225, Thermo Fischer Scientific). Protein from the cell lysates was precipitated in 50% Trichloracetic acid with 1% BSA, overnight at −20°C, followed by centrifugation. The protein pellet was then washed in acetone, dissolved in 0.5 mol L^−1^ NaOH and the amount of [^14^C] determined by scintillation counting (WinSpectral 1414 Liquid Scintillation Counter; Wallac, Turku, Finland). Counts per minute were normalized to the total amount of protein per well, and the amount of phenylalanine incorporated into protein is presented as pmol mg^−1^ h^−1^.

### [^3^H] Palmitic acid oxidation assay

[^3^H] Palmitic acid oxidation was measured by production of [^3^H] labeled water as previously described (Rune et al. 2009). Differentiated myotubes were incubated for 6 h in DMEM (1 g L^−1^ glucose) with 1 mCi mL^−1^ [^3^H] palmitic acid (#NET043005MC, Perkin Elmer), 25 *μ*mol L^−1^ palmitic acid and 0.02% fatty acid free BSA. The collected medium was incubated in a charcoal slurry (0.1 g activated charcoal per 1 mL 0.02 mol L^−1^ Tris‐HCl at pH 7.5), subjected to centrifugation, and the supernatant was collected for scintillation counting (WinSpectral 1414 Liquid Scintillation Counter; Wallac). Cells were lysed in 0.03% SDS and the protein concentration was determined using a commercially available Bradford protein assay kit (#5000006, BioRad). Results were normalized to the total protein amount per well and the oxidation rate of palmitic acid is presented as pmol mg^−1^ h^−1^.

### Statistical analysis

**Figure 1:**
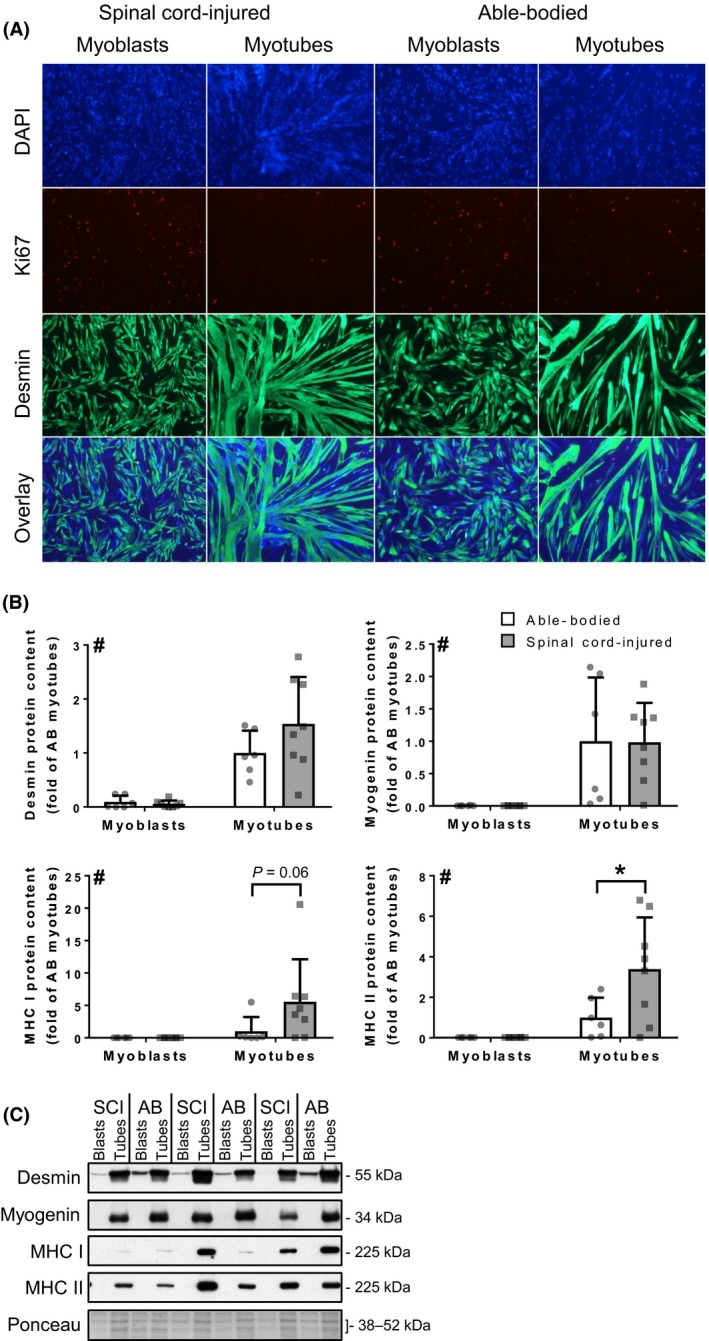
Differentiation of skeletal muscle satellite cells. (A) Representative images of immunohistochemistry for Ki67 and desmin, as well as DAPI staining of the nuclei and an overlay of the three signals. (B) Protein content of muscle‐specific differentiation markers (desmin, myogenin, MHC I and MHC II) in myoblasts and myotubes from spinal cord‐injured (gray bars) and able‐bodied (white bars) individuals. Bars represent mean ± SD and individual data points are overlaid. *n* = 6–8; Two‐way ANOVA: # – overall effect of differentiation (*P* < 0.05); * – Sidak's post hoc comparison (*P* < 0.05). (C) Representative Western blot images. DAPI, 4,6‐diamidino‐2‐phenylindole; MHC, myosin heavy chain.

**Figure 2:**
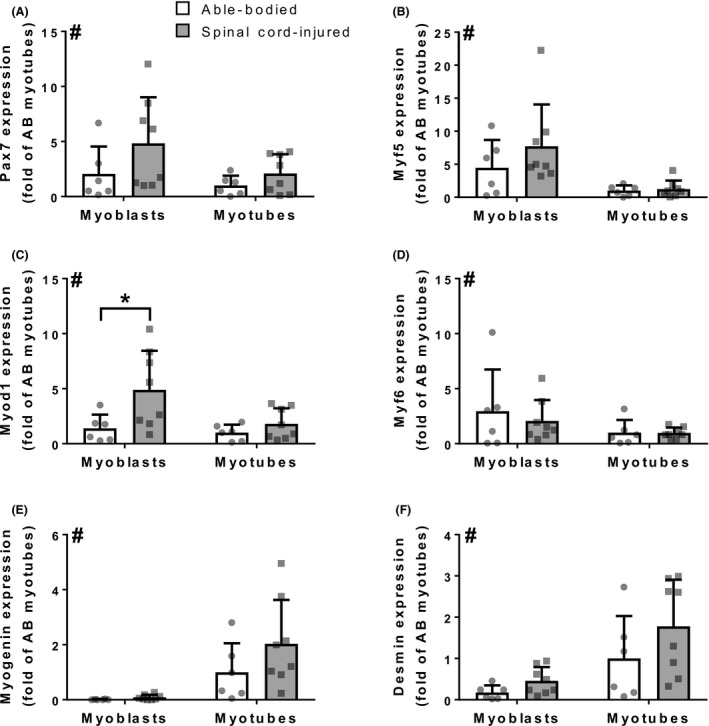
Regulation of differentiation of skeletal muscle satellite cells. Expression of myogenic regulatory factors (A) Pax7, (B) Myf5, (C) Myod1, (D) Myf6, (E) myogenin, and (F) desmin, in myoblasts and myotubes from spinal cord‐injured (gray bars) and able‐bodied (white bars) individuals. Bars represent mean ± SD and individual data points are overlaid. *n* = 6–8; Two‐way ANOVA: # – overall effect of differentiation (*P* < 0.05); * – Sidak's post hoc comparison (*P* < 0.05).

**Figure 3:**
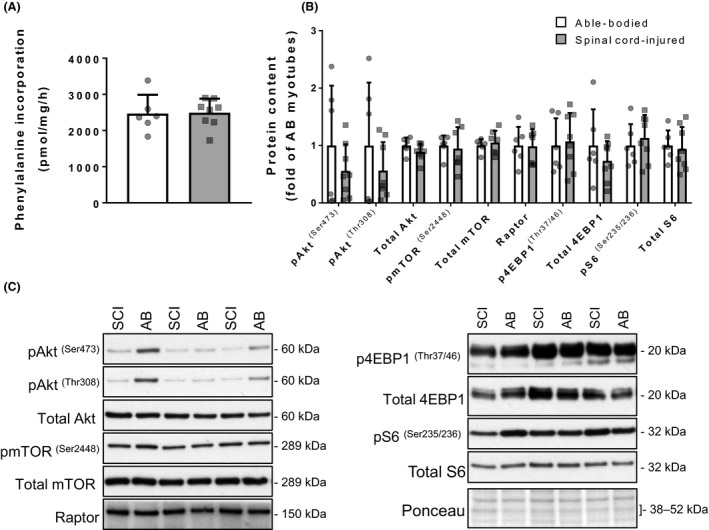
Protein synthesis in differentiated myotubes. (A) Phenylanine incorporation into protein in myotubes from spinal cord‐injured (gray bars) and able‐bodied (white bars) individuals. Bars represent mean ± SD and individual data points are overlaid. (B) Phosphorylated and total protein content of molecules in the Akt‐mTOR signalling axis (pAkt^(Ser473)^, pAkt^(Thr308)^, total Akt, mTOR ^(Ser2448)^, total mTOR, Raptor, p4EBP1^(Ser37/46)^, total 4EBP, pS6^(Ser235/236)^, total S6) in myotubes from spinal cord‐injured (gray bars) and able‐bodied (white bars) individuals. Bars represent mean ± SD and individual data points are overlaid. *n* = 6–8; Mann–Whitney test (significance *P* < 0.05). (C) Representative Western blot images. mTOR, mechanistic target of rapamycin; 4EBP, 4E binding protein.

**Figure 4:**
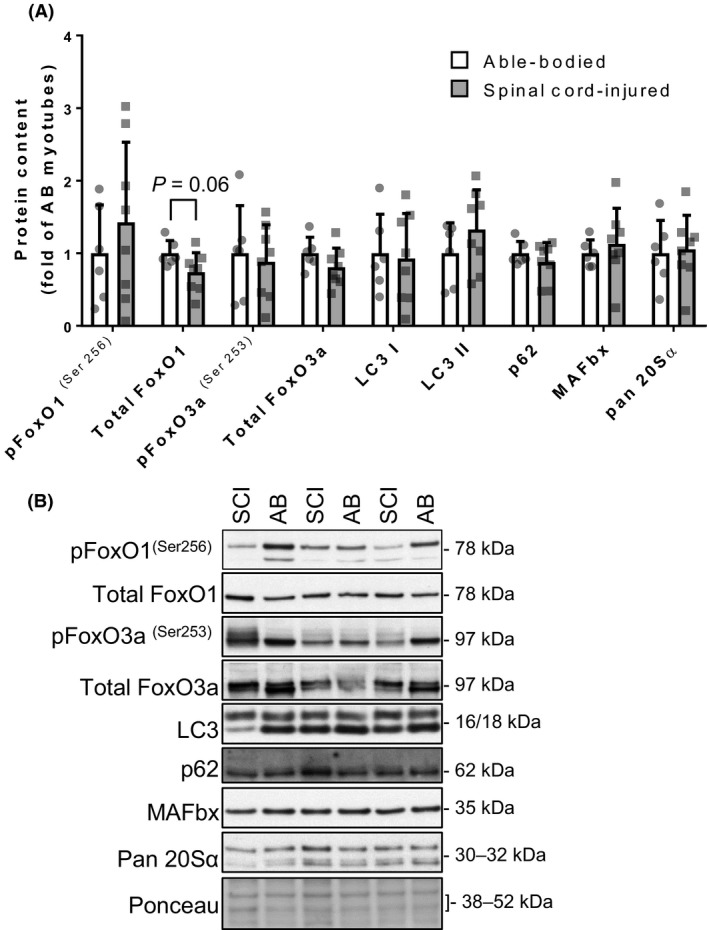
Protein degradation signalling in differentiated myotubes. (A) Phosphorylated and total protein content of FoxO1^(Ser256)^ and FoxO3^(Ser253)^ transcription factors, and their targets (LC3I, LC3II, p62, MAFbx), as well as the total protein levels of 20S*α* proteosomal subunit in myotubes from spinal cord‐injured (gray bars) and able‐bodied (white bars) individuals. Bars represent mean ± SD and individual data points are overlaid. *n* = 6–8; Mann–Whitney test (significance *P *<* *0.05). (B) Representative Western blot images. FoxO, forkhead box protein O; LC, light chain; MAFbx, muscle atrophy F‐box.

**Figure 5:**
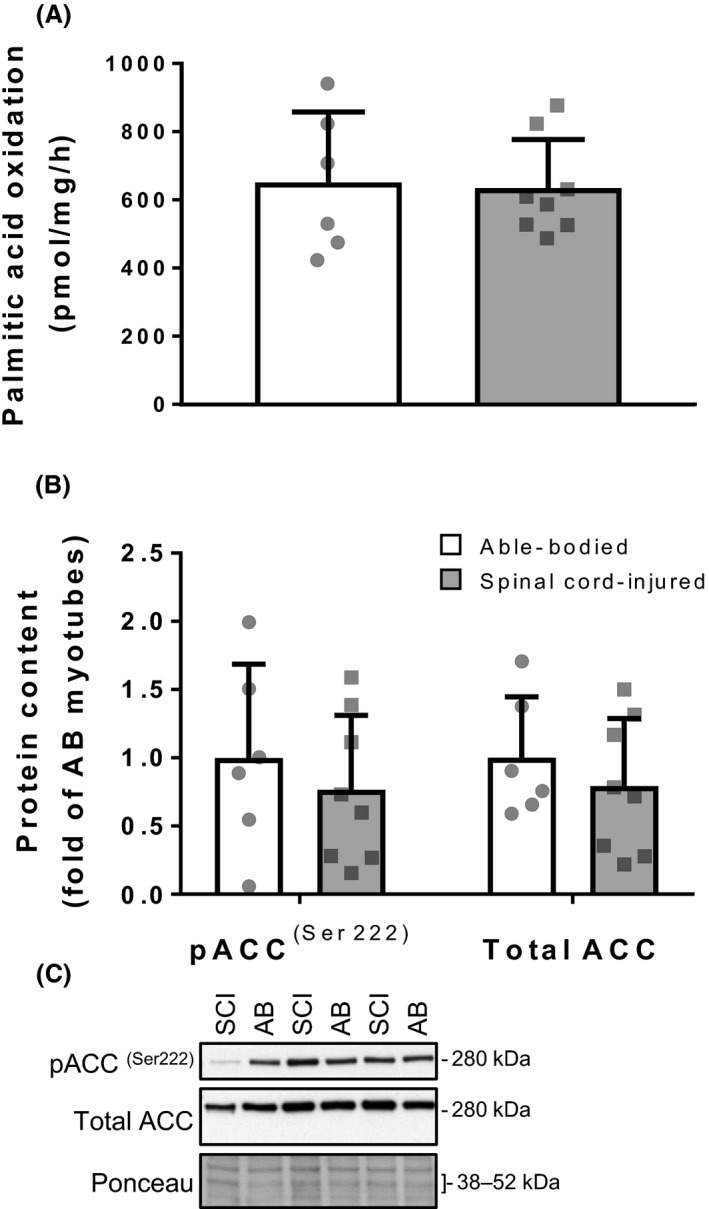
Oxidative capacity of differentiated myotubes. (A) Palmitic acid oxidation in myotubes from spinal cord‐injured (gray bars) and able‐bodied (white bars) individuals. Bars represent mean ± SD and individual data points are overlaid. (B) Protein content of total and phosphorylated ACC ^(Ser222)^ in myotubes from spinal cord‐injured (gray bars) and able‐bodied (white bars) individuals. Bars represent mean ± SD and individual data points are overlaid. *n* = 6–8; Mann–Whitney test: (significance *P *<* *0.05). (C) Representative Western blot images. ACC, acetyl‐CoA carboxylase.

Statistical significance of differences in gene expression and protein content in myoblasts and myotubes from spinal cord‐injured and able‐bodied individuals were determined by a two‐way ANOVA, followed by the Sidak's corrected multiple comparison test (Figs. 1, 2). Differences between myotubes of the two groups were determined by the Mann–Whitney test (Figs. 3, 4, 5). *P* values below 0.05 were considered as statistically significant, while values below 0.1 are reported as trends. Statistical analyses were performed, using the GraphPad Prism v. 7.01 (GraphPad, La Jolla, CA).

## Results

### Differentiation of skeletal muscle satellite cells

To determine the differentiation capacity of skeletal muscle satellite cells, activated satellite cells (myoblasts) were differentiated into myotubes. We assessed myotube formation by immunohistochemistry for differentiation (desmin) and proliferation (Ki67) markers and nuclear staining with DAPI. Qualitative assessment of immunohistochemistry images has shown that, upon differentiation, myoblasts from both spinal cord‐injured and able‐bodied individuals fused and formed multinucleated myotubes with an abundance of desmin and depletion of Ki67 positive nuclei (Fig. 1A).

Western blot was used to assess protein levels of differentiation markers of myoblasts and myotubes. Differentiation increased (*P* < 0.05) cellular desmin, myogenin and both type I and type II myosin heavy chain (MHC I and II; Fig. 1B and C, respectively). Myotubes from spinal cord‐injured and able‐bodied individuals had similar (*P* > 0.05) levels of desmin and myogenin. Myotubes from spinal cord‐injured individuals displayed higher (*P* < 0.05) protein abundance of MHC II, whereas MHC I tended (*P* = 0.06) to be increased (Fig. 1B and C).

We next determined whether spinal cord injury affects the transcriptional regulation of differentiation by assessing the expression of myogenic regulatory factors in myoblasts and myotubes from spinal cord‐injured and able‐bodied individuals. The expression of Pax7, Myf5, Myod1 and myogenic factor 6 (Myf6) decreased (*P* < 0.05) throughout differentiation (Fig. 2A–D). Conversely, the expression of myogenin and desmin was increased (*P* < 0.05) in myotubes compared to myoblasts (Fig. 2E and F). Expression of Pax7, Myf5, Myf6, myogenin and desmin was similar (*P* > 0.05) in myoblasts, as well as in myotubes from the spinal cord‐injured and able‐bodied individuals (Fig. 2A, B and D–F; *P* > 0.05). Expression of Myod1 was higher (*P* < 0.05) in myoblasts from spinal cord‐injured individuals compared to able‐bodied controls (Fig. 2C).

### Protein synthesis in differentiated myotubes

To determine whether myotubes from spinal cord‐injured individuals have an altered capacity for protein synthesis we measured incorporation of [^14^C]‐labeled phenylalanine into protein. The incorporation of radiolabeled tracer into protein was similar (*P* > 0.05) between myotubes from spinal cord‐injured and able‐bodied individuals (Fig. 3A). We found similar (*P* > 0.05) levels of phosphorylated Akt^(Ser473)^ and ^(Thr308)^, mTOR^(Ser2448)^, 4E binding protein 1 (4EBP1)^(Thr37/46)^, ribosomal protein S6 (S6)^(Ser235/236)^, and the corresponding total protein content in spinal cord‐injured and able‐bodied individuals (Fig. 3B and C). Similarly, content of regulatory‐associated protein of mTOR (raptor) was not different (*P* > 0.05) between myotubes from spinal cord‐injured and able‐bodied individuals (Fig. 3B and C).

### Protein degradation signalling in differentiated myotubes

We used Western blot analysis to investigate pathways regulating protein degradation. Phosphorylated FoxO1^(Ser256)^ and FoxO3a^(Ser253)^, as well as the total abundance of FoxO3a were similar (Fig. 4A and B; *P* > 0.05) between spinal cord‐injured and able‐bodied individuals. Total protein content of FoxO1 tended to be decreased (Fig. 4A and B; *P* = 0.06) in myotubes from spinal cord‐injured compared to able‐bodied individuals. Protein levels of the FoxO transcriptional targets including ubiquitin‐binding protein p62 (p62), microtubule‐associated protein 1 light chain 3 (LC3) I and II, and muscle atrophy F‐box (MAFbx) were unchanged (Fig. 4A and B; *P* > 0.05). Similarly, protein abundance of the proteasomal 20S*α* catalytic subunit was comparable between myotubes from spinal cord‐injured and able‐bodied individuals (Fig. 4A and B; *P* > 0.05).

### Fatty acid oxidation in differentiated myotubes

To determine the oxidative capacity of myotubes derived from satellite cells of spinal cord‐injured individuals, we measured palmitic acid oxidation by production of [^3^H]‐labeled water from [^3^H]‐labeled palmitic acid. The oxidation of palmitic acid was similar between satellite cells of spinal cord‐injured and able‐bodied individuals (*P* > 0.05; Fig. 5A). Moreover, total and phosphorylated protein content of acetyl‐CoA carboxylase (ACC)^(Ser222)^ was similar (*P* > 0.05) between skeletal muscle satellite cells of spinal cord‐injured and able‐bodied individuals (Fig. 5B and C).

## Discussion

Satellite cells play an important role in skeletal muscle regeneration and plasticity (Schiaffino et al. 1976; Collins et al. 2005; Bruusgaard et al. 2010; Lepper et al. 2011). However, little is known regarding their differentiation capacity in human skeletal muscle following a spinal cord injury. We assessed the myogenic programming of satellite cells obtained from skeletal muscle of spinal cord‐injured individuals. We provide evidence that skeletal muscle satellite cells retain their capacity to differentiate after spinal cord injury. Moreover, once differentiated the myotubes show comparable metabolic characteristics to those from able‐bodied individuals. Collectively, our data indicates that the intrinsic myogenic programming of skeletal muscle satellite cells is retained in individuals with spinal cord injury.

During satellite cell isolation, we attempted to filter out nonsatellite cells by allowing their attachment, although a formal assessment of purity was not performed. Our study included spinal cord‐injured participants after a longstanding (>1 year) injury, a period when skeletal muscle has undergone dramatic skeletal muscle atrophy (Moore et al. 2015). We show that satellite cells do not mirror such prominent changes when grown in vitro. However, due to the relatively low number of participants (*n* = 6–8), it is possible that more subtle changes exist which we are underpowered to detect. Finally, the spinal‐cord injury group consisted of participants with both incomplete and complete spinal‐cord injury, but none were freely ambulating. However, we cannot rule out that remaining motor activity or muscular spasms may influence satellite cell function.

We have shown that during differentiation, myoblasts from spinal cord‐injured individuals retain the capacity to exit the cell cycle, as evidenced by the decrease of Ki67 nuclear localization through differentiation (Scholzen and Gerdes 2000), and form multinucleated myotubes, with levels of desmin similar to myotubes from able‐bodied controls. Additionally, protein abundance of muscle specific structural proteins (desmin and MHC) was increased during differentiation in both cells from able‐bodied and spinal cord‐injured individuals. Surprisingly, we found the abundance of MHC I and MHC II was increased in myotubes from spinal cord‐injured versus able‐bodied individuals. Spinal cord injury is associated with a decrease of MHC I and an increase of MHC II in skeletal muscle in vivo, reflecting the change in fiber type‐composition from type I to predominantly type II fibers (Lotta et al. 1991; Castro et al. 1999; Kostovski et al. 2013). Our findings of increased MHC II in differentiated myotubes from spinal cord‐injured individuals may indicate that satellite cells retain a fiber‐type memory. However, we found a trend (*P* = 0.06) for increased MHC I in myotubes from spinal cord‐injured subjects, suggesting that the alteration in the MHC content in myotubes may be caused by different mechanisms. Nevertheless, the capacity to increase skeletal muscle‐specific structural proteins during differentiation indicates that the differentiation capacity in satellite cells from spinal cord‐injured individuals is preserved.

Pax7 and myogenic regulatory factors direct differentiation (Almeida et al. 2016). In the early stages of differentiation, commitment to the myogenic lineage is evident by high expression of Myf5 and Myod1 (Almeida et al. 2016). Distinct populations of myoblasts express either Myf5 or Myod1 upon activation, and these factors show an overlapping role in differentiation (Rudnicki et al. 1993; Cooper et al. 1999). As myoblasts from spinal cord‐injured individuals had higher Myod1 expression compared to those from able‐bodied controls, a larger portion of myoblasts from spinal cord‐injured individuals may belong to the Myod1 positive group. As differentiation progresses, the terminal stages are characterized by increased myogenin expression (Almeida et al. 2016). In cells from both able‐bodied and spinal cord‐injured individuals, the expression of Pax7, Myf5 and Myod1 decreased through differentiation, while the expression of myogenin increased through differentiation. These data suggest that satellite cells from spinal cord‐injured individuals are committed to the myogenic lineage, evidenced by high Myf5 and Myod1 expression, and are able to undergo terminal differentiation and increase myogenin expression in myotubes. Thus, in addition to their differentiation capacity, the regulation of differentiation in satellite cells from spinal cord‐injured individuals is preserved. In the current study, we have not determined the proliferation capacity of satellite cells. As spinal cord injury leads to their activation (Dupont‐Versteegden et al. 1999; Jayaraman et al. 2013), this is a parameter of interest, especially if inherent satellite cells are to be used to combat muscle atrophy.

Loss of skeletal muscle mass is mainly attributed to an imbalance between anabolic and catabolic protein metabolism (Jackman and Kandarian 2004). Akt‐mTOR signalling undergoes a coordinated decrease in skeletal muscle of spinal cord‐injured in vivo, indicating decreased protein synthesis (Dreyer et al. 2008). Our findings of unchanged protein abundance of the Akt‐mTOR signalling axis members and amino acid incorporation into protein in myotubes directly contrast the in vivo observations. We conclude that skeletal muscle satellite cells after spinal cord injury retain their ability to produce myotubes with a normal capacity for, and regulation of protein synthesis.

With chronic spinal cord injury, FoxO transcriptional activity and the expression of their downstream targets involved in protein degradation are reduced (Leger et al. 2009; Milan et al. 2015). We reported a tendency (*P* = 0.06) for reduced total FoxO1 protein in myotubes from spinal cord‐injured individuals, with no changes in phosphorylated (inactive) levels of both FoxO1^(Ser256)^ and FoxO3a^(Ser253)^, as well as the total protein content of FoxO3a. The tendency of reduced FoxO1 total protein content could indicate lower transcriptional activity. However, as there were no changes in protein abundance of FoxO transcriptional targets (p62, LC3I, LC3II and MAFbx) (Milan et al. 2015) we conclude that in contrast to the observations in skeletal muscle in vivo following spinal cord injury, the FoxO transcriptional regulation of protein degradation is unchanged in satellite cell‐derived myotubes in vitro.

Additionally, the comparable levels of p62, the ubiquitin‐binding autophagy cargo adapter, and LC3I and II, the autophagosome‐forming proteins, indicate similar levels of macroautophagy between myotubes from spinal cord‐injured and able‐bodied individuals (Mizushima and Yoshimori 2007). Unchanged abundance of MAFbx, an ubiquitin E3 ligase and pan20S*α* proteasomal proteolytic subunit in myotubes from spinal cord‐injured individuals, indicate stable levels of ubiquitination and proteosomal degradation, respectively. Together, this could indicate stable levels of protein degradation in myotubes from spinal cord‐injured individuals.

Skeletal muscle following spinal cord injury in vivo has decreased *β*‐oxidation, mirrored by reductions in free fatty acid uptake, mitochondrial content and levels of oxidative enzymes (Wang et al. 1999; Kjaer et al. 2001; Long et al. 2011; McCully et al. 2011). Conversely, myotubes from spinal cord‐injured and able‐bodied individuals were able to oxidize palmitic acid at a comparable level. Total and phosphorylated protein content of ACC^(Ser222)^ were similar between the two groups, indicating stable regulation of the fatty acid metabolism. Thus, in contrast to the reduced *β*‐oxidation capacity in vivo, skeletal muscle satellite cells from spinal cord‐injured individuals are able to produce myotubes with oxidative capacity comparable to those from able‐bodied controls. Collectively, our data shows that the metabolic memory of satellite cells is retained and they are able to produce myotubes with normal protein and fatty acid metabolism, in spite of the changes occurring in skeletal muscle in vivo.

Previous animal studies indicate that spinal cord injury leads to activation of satellite cells in the affected skeletal muscle in vivo (Dupont‐Versteegden et al. 1999; Jayaraman et al. 2013). However, the terminal differentiation of the cells may be lacking as the myonuclear number continues to decrease (Dupont‐Versteegden et al. 1999). Other rat models of skeletal muscle atrophy, such as lower motor neuron injury and denervation, also lead to activation of satellite cells followed by inefficient differentiation and underdeveloped myotubes, with deficient or absent contractile machinery (Carraro et al. 2015). Similar mechanisms, through activation and inefficient differentiation, may be responsible for the reduction of the satellite cell pool in the skeletal muscle of spinal cord‐injured individuals (Verdijk et al. 2012). However, our data demonstrates that the intrinsic myogenic differentiation capacity and the metabolic memory of satellite cells from spinal cord‐injured individuals are preserved following spinal cord injury. Once extracted from the skeletal muscle and grown in vitro, they differentiate and produce myotubes that retain metabolic characteristics. Thus, defects in satellite cell differentiation in skeletal muscle of spinal cord‐injured individuals may be connected to the decentralized and atrophying skeletal muscle “environment”, rather than a dysfunction in their programming.

As satellite cells play a role in regulating skeletal muscle mass (Bruusgaard et al. 2010), specific rehabilitative interventions targeting their activation could be efficient in reducing skeletal muscle atrophy after spinal cord injury. Electrical stimulation coupled with exercise improves the metabolic characteristics of skeletal muscle in spinal cord‐injured individuals (Hjeltnes et al. 1998; Gorgey et al. 2017) and may be used as a potential activator of skeletal muscle regenerative machinery (Kern and Carraro 2014). Different protocols of functional electrical stimulation mimic different types of exercise (Fornusek and Davis 2008; Bickel et al. 2015). Both endurance and resistance training lead to an increase in satellite cell number, while resistance training also leads to an increase in myonuclear number (Bruusgaard et al. 2010; Kurosaka et al. 2012). In light of this, studies of satellite cell response to different modalities and intensities of functional electrical stimulation could help design specialized interventions for satellite cell activation. Further studies analysing the response to electrical pulse stimulation in vitro could reveal the full extent of functionality of myotubes and inform these efforts in regard to any underlying electrical stimulus intensity, frequency and duration preference. Our findings may encourage these future efforts, and highlight the prospect of inherent satellite cell activation in attempts to prevent skeletal muscle atrophy following spinal cord injury.

## Conflict of Interest

No conflicts of interest are declared by the authors.

## Data Accessibility
